# Fibronectin Binding Modulates CXCL11 Activity and Facilitates Wound Healing

**DOI:** 10.1371/journal.pone.0079610

**Published:** 2013-10-25

**Authors:** Federico Tortelli, Marco Pisano, Priscilla S. Briquez, Mikaël M. Martino, Jeffrey A. Hubbell

**Affiliations:** 1 Institute of Bioengineering, School of Life Sciences and School of Engineering, École Polytechnique Fédérale de Lausanne, Lausanne, Switzerland; 2 Institute of Chemical Sciences and Engineering, School of Basic Sciences, École Polytechnique Fédérale de Lausanne, Lausanne, Switzerland; Instituto de Engenharia Biomédica, University of Porto, Portugal

## Abstract

Engineered biomatrices offer the potential to recapitulate the regenerative microenvironment, with important implications in tissue repair. In this context, investigation of the molecular interactions occurring between growth factors, cytokines and extracellular matrix (ECM) has gained increasing interest. Here, we sought to investigate the possible interactions between the ECM proteins fibronectin (FN) and fibrinogen (Fg) with the CXCR3 ligands CXCL9, CXCL10 and CXCL11, which are expressed during wound healing. New binding interactions were observed and characterized. Heparin-binding domains within Fg (residues 15-66 of the β chain, Fg β15-66) and FN (FNI1-5, but not FNIII12-14) were involved in binding to CXCL10 and CXCL11 but not CXCL9. To investigate a possible influence of FN and Fg interactions with CXCL11 in mediating its role during re-epithelialization, we investigated human keratinocyte migration *in vitro* and wound healing *in vivo* in diabetic *db/db* mice. A synergistic effect on CXCL11-induced keratinocyte migration was observed when cells were treated with CXCL11 in combination with FN in a transmigration assay. Moreover, wound healing was enhanced in full thickness excisional wounds treated with fibrin matrices functionalized with FN and containing CXCL11. These findings highlight the importance of the interactions occurring between cytokines and ECM and point to design concepts to develop functional matrices for regenerative medicine.

## Introduction

Regenerative medicine has evolved from an initial focus on transplanted cells to also embrace development of advanced biomatrices laden with active biomolecules that enhance tissue repair and control the host response by mimicking the regenerative microenvironment[1,2]. In this context, a full comprehension of the overall signaling network driving wound healing is still missing and the complex pattern of interactions between the different biological moieties involved (i.e. cytokines, growth factors, extracellular matrix (ECM) proteins, integrin ligands) is incompletely understood. Still, new insights into the pivotal interactions regulating dermal wound healing can have important implications for new therapies, such as in treatment of diabetic foot ulcers.

Skin wound healing is the result of a complex network of biological events where the ECM and other soluble molecules such as growth factors and cytokines play a fundamental role in driving the fate of different cell types. Recently, an important role has become clear for the ELR-negative cytokines CXCL10 and CXCL11, signaling through their common receptor CXCR3, in coordinating the regenerative and resolving phase of cutaneous healing[3]. Delivery of a CXCL11 antisense construct worsened wound healing, leading to delayed re-epithelialization and impaired epidermis maturation, a phenotype observed in CXCR3-deficient mice as well[4-6]. Although a full understanding of the biological processes linking the CXCR3 axis to wound healing is still missing, it is now clear that CXCL11, expressed in injured epidermis, plays an important role in enhancing undifferentiated keratinocyte motility, thus coordinating the resolution and regenerative phase together with signals coming from the ECM [3,7]. 

Recently, the importance of the ECM in presenting growth factors during the regenerative phase of wound healing has gained interest, particularly in view of development of new biomatrices for morphogen delivery[8,9]. Interactions between ECM proteins and morphogens modulate local morphogen concentration and signaling and can create specific biomolecular gradients based on the ECM composition. Good examples are the interactions between fibroblast growth factor-2 (FGF-2), transforming growth factor beta (TGF-β) and vascular endothelial growth factor-A (VEGF-A) with proteoglycans as well as ECM proteins vitronectin, fibrin and FN[10-15]. Heparin binding domains of different ECM proteins have been shown to bind certain growth factors. In recent studies, our laboratory has shown rather promiscuous growth factor interactions with the 12^th^-14^th^ type III repeats of fibronectin (FNIII 12-14)[10], residues 15-66 of the β chain of fibrinogen (Fg β15-66)[16] and the 5th fibronectin-like repeat of tenascin C[17]. Such interactions were engineered to develop biomatrices for tissue repair through presentation of growth factors such as VEGF-A and PDGF-BB [9,16].

Here, we sought to investigate potential interactions occurring between two of the most important matrix proteins, FN and Fg, and CXCR3 ligands expressed in injured epidermis, CXCL9, CXCL10 and CXCL11[18]. Discovering such binding with CXCL10 and CXCL11, we turned our attention to the effects of these interactions both *in vitro* and *in vivo* in the context of the role of CXCL11 in modulating keratinocyte migration and coordinating re-epithelialization. We show that the interaction of CXCL11 with FN can be exploited in fibrin matrices to enhance wound healing in the *db/db* diabetic mouse. 

## Results

### Fibronectin and fibrinogen bind CXCL10 and CXCL11, but not CXCL9

**Figure 1:**
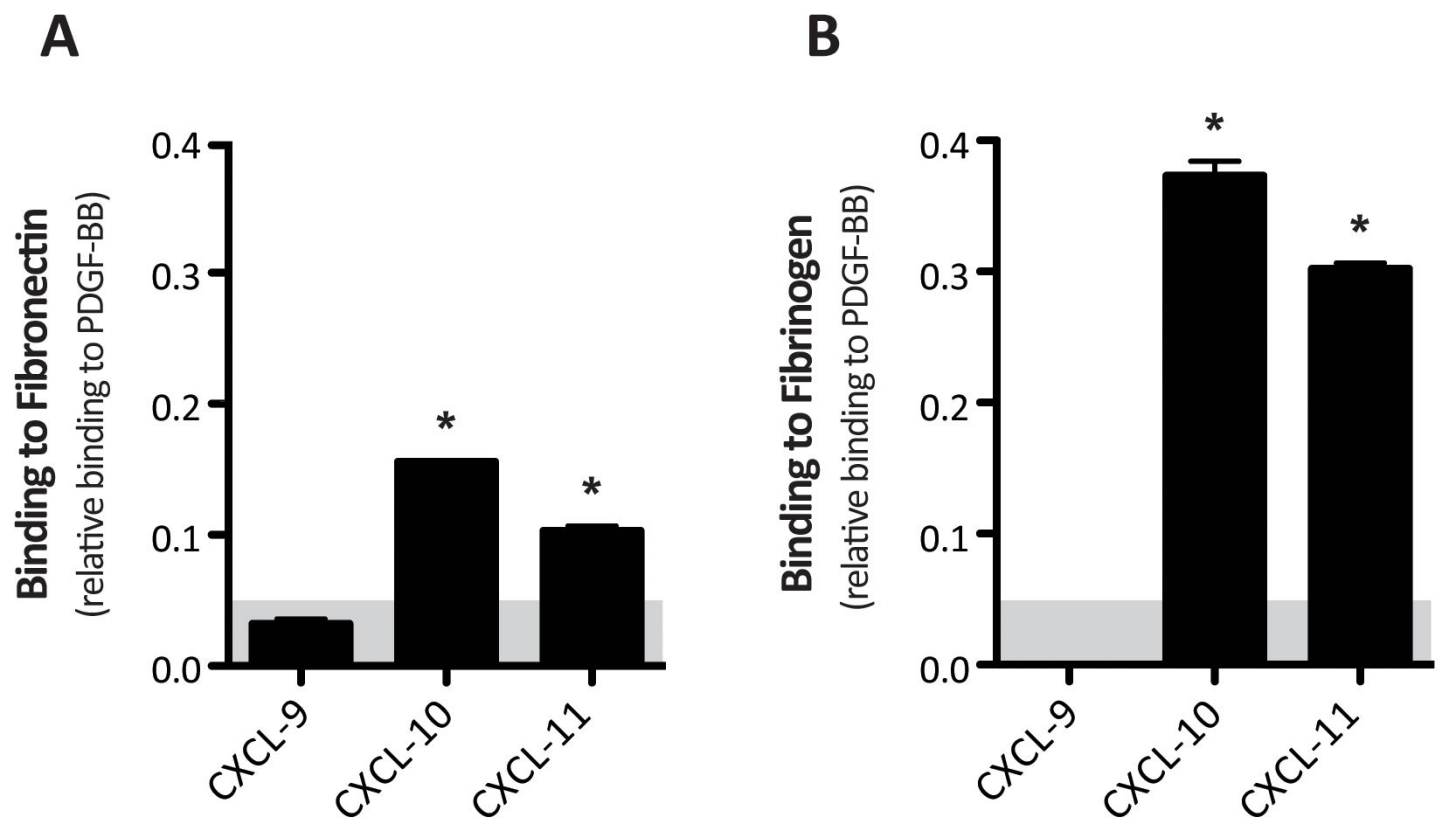
Fibronectin and fibrinogen binding to CXCL9, CXCL10 and CXCL11. CXCL9, CXCL10 and CXCL11 binding to FN (A) and Fg (B) was determined by indirect ELISA and calibrated to PDGF-BB binding to FN as a strongly binding reference (Abs 450nm = 0.59 AU) and Fg (Abs 450 nm=0.79 AU), respectively. FN and Fg binding to BSA were considered as background and subtracted. Binding of CXCL10 and CXCL11, but not CXCL9, was observed (*). (n=6, mean ± SD).

We first investigated the ability of FN and Fg to bind CXCL9, CXCL10 and CXCL11, using an indirect ELISA. PDGF-BB was used as positive control due to its high affinity binding to both of these ECM molecules [10,16], while binding to bovine serum albumin (BSA) was measured as negative reference control. Briefly, the cytokines were coated on ELISA plates, blocked and subsequently incubated with either FN or Fg. Binding was evaluated by using a specific antibody against either FN or Fg and normalized to binding on adsorbed PDGF-BB. Since weak binders of FN such as PDGF-CC and VEGF-C were previously reported to bind FN with a relative binding to PDGF-BB close to 0.05 we define this value as threshold to define relevant binders [10]. CXCL10 and CXCL11 were both observed to bind FN and Fg, while CXCL9 was not (Figure 1). Binding to other cytokines from the CC chemokine, interferon and interleukin families was also investigated. Among interleukins, only IL-2 and IL-4 weakly bound to FN (Figure S1), and IL-1α was the only interleukin showing a positive signal when tested for Fg binding (Figure S2). Among the members of interferon family, IFN-γ showed binding to both FN and Fg with high affinity, while IFN-β was only a weak binder of Fg (Figure S1, Figure S2). In the CCL family, CCL-20 showed a low level of binding to FN (Figure S1). Importantly, since our group previously reported no differences in coating efficiencies between growth factors coming from the same family[10,16], the lack of signal shown by some cytokines likely was not related to poor coating efficiency on the ELISA plate. 

### FNI 1-5 and Fg β15-66 are involved in binding of fibronectin and fibrinogen to CXCL10 and CXCL11

**Figure 2:**
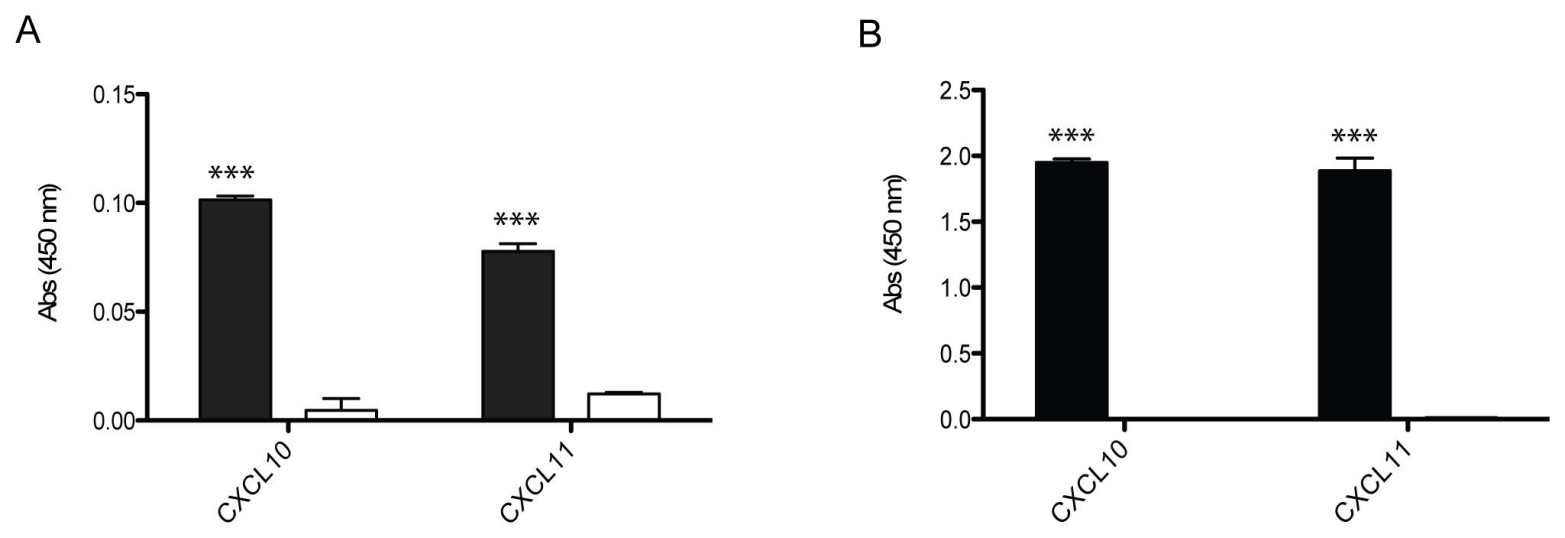
FNI 1-5 and Fg β15-66 binding. FNI 1-5 and Fg β15-66 binding to CXCL10 and CXCL11 was determined by indirect ELISA. ELISA plates were coated with either CXCL10 or CXCL11 and probed with either FNI 1-5 (black bars) (A) or Fg β15-66 (black bars) (B). Binding to BSA was measured as negative control (white bars) (n=3, mean ± SD, Student’s t-test, *p<0.001).

**Figure 3:**
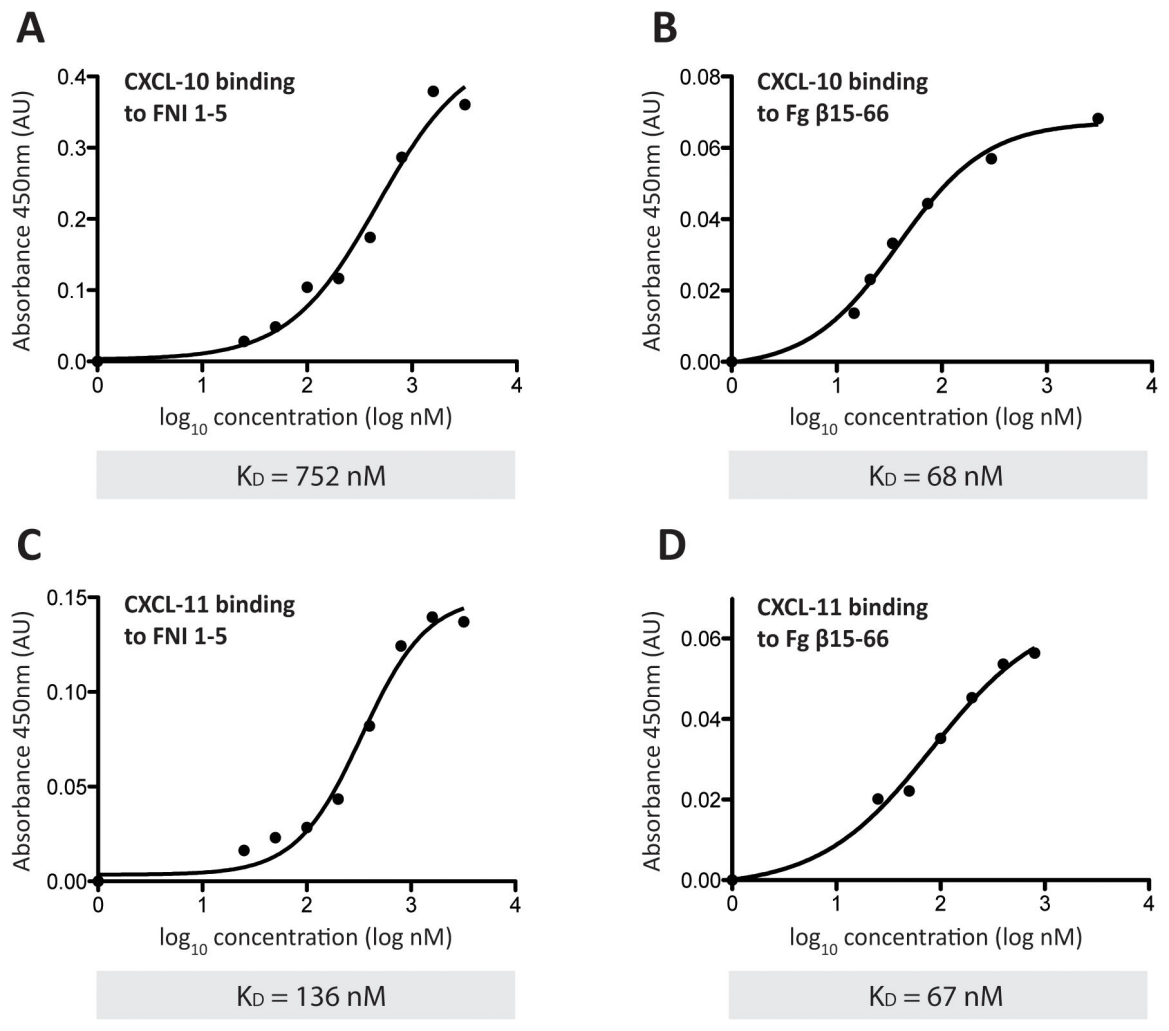
K_D_ analysis. K_D_ was determined by indirect ELISA. ELISA plates were coated with CXCL10 (A, B) or CXCL11 (C, D) and probed with increasing concentrations (25 nM-3.2 μM) of either FNI 1-5 (A, C) or Fg β15-66 (B, D) in the absence of heparin. Binding to BSA was considered as background and subtracted (n=3, mean).

Since heparin-binding domains of FN and Fg have already been described as promiscuous growth factor binding domains involved in interactions between ECM molecules and morphogens [9,16,17], we sought to investigate the possible role of their possible role in binding to CXCL10 and CXCL11. FNIII 12-14, the 1st-5th type I repeats of FN (FNI 1-5) and the residues Fg β15-66 were recombinantly produced and tested for binding to cytokines. While the tested cytokines did not bind to FNIII 12-14 (data not shown), they showed a significant affinity to FNI 1-5 and Fg β15-66 by indirect ELISA (Figure 2), with BSA being used as negative control. To better evaluate the affinity of binding to both of these heparin-binding domains, the equilibrium binding constants (K_d_) were determined by ELISA as described elsewhere[19]. In general, Fg β15-66 was able to bind the two cytokines with higher affinity than FNI 1-5. Indeed, K_d_ values of 67 and 68 nM were observed for the binding of Fg β15-66 to CXCL11, CXCL10, respectively. Higher K_d_ values of 136 nM and 752 nM were observed for binding of FNI 1-5 to the same cytokines (Figure 3). Student’s t-test was performed in order to confirm FNI 1-5 and Fg β15-66 binding. 

### CXCL10 and CXCL11 binding to FNI 1-5 and Fg β15-66 is inhibited by heparin

**Figure 4:**
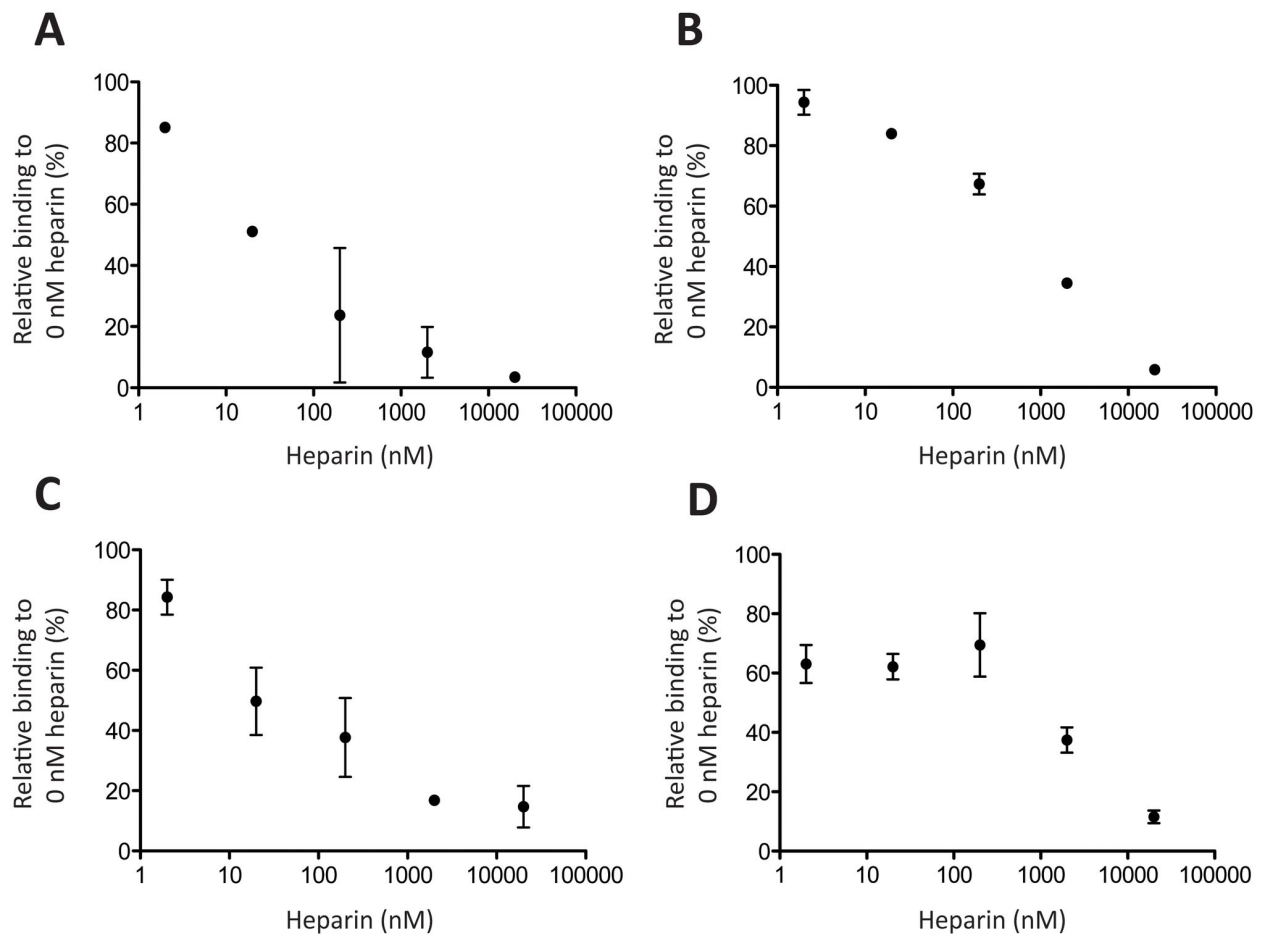
Heparin competition analysis. ELISA plates were coated with CXCL10 (A, B) or CXCL11 (C, D) and probed with FNI 1-5 (A, C) or Fg β15-66 (B, D) in the presence of increasing concentrations of heparin (2 nM - 20 μM). Binding was related to the binding observed in the absence of heparin. Binding to BSA was considered as background and subtracted. (n=3, mean±SD).

To investigate the possible role of heparin in regulating cytokine binding, the same indirect ELISA method described above was performed in presence of different heparin concentrations (2 nM-20 μM). CXCL10 and CXCL11 binding to FNI 1-5 and Fg β15-66 was inhibited by the presence of heparin at low and high concentrations (Figure 4). In general, heparin reduced binding to both ECM domains, and a high excess of heparin, at 2 μM, essentially blocked the binding of the cytokines to the evaluated heparin binding domains (Figure 4). 

### CXCL11-induced keratinocyte migration is enhanced in presence of fibronectin

**Figure 5:**
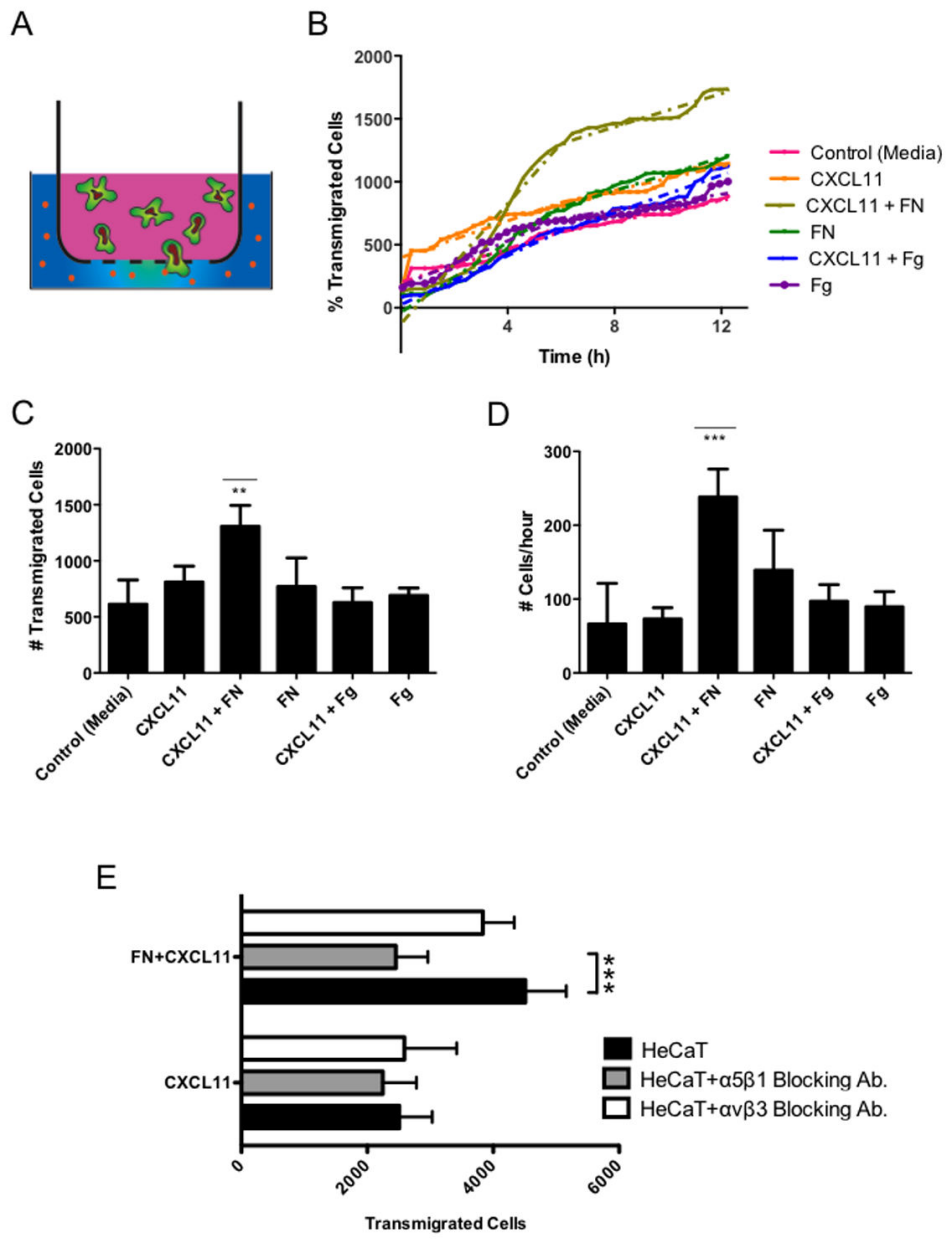
*Keratinocyte migration* towards CXCL11. *In* *vitro* keratinocyte migration was evaluated by a modified Boyden chamber transmigration assay. HeCaT cells were stained with Vybrant® DIO cell tracer and seeded in the upper chamber. The number of cells transmigrating towards the test medium contained in the lower chamber was monitored during the time of the experiment by live imaging microscopy (A). Transmigration was quantified through automatic cell identification and cell counting within 12 hr. Solid lines show the number of migrating cells. Dashed lines show segmented interpolation analysis of the curves. (B). Number of transmigrated HeCaT cells (C) and transmigration speed (D) during the first 6 hr of the assay. (E) Number of transmigrated HeCaT cells in presence of blocking antibodies for α5β1 and αvβ3 integrins. (n=6, mean ± SD, One-way ANOVA with Dunnett’s comparison to Control, ** p<0.01, *** p<0.001).

Since CXCL11 has an important role during re-epithelialization by coordinating keratinocyte migration, we sought to investigate whether the interactions between CXCL11, FN and Fg could modulate this biological process *in vitro*. HeCaT cells (immortalized human keratinocytes) transmigration towards CXCL11 in the presence of either FN or Fg was analyzed by a modified Boyden chamber transmigration assay (Figure 5A). Briefly, HeCaT cells were seeded in the upper chamber and the number of cells transmigrating towards the testing medium contained in the lower chamber was monitored during the time of the experiment. In general, CXCL11-driven keratinocyte migration was strongly enhanced when CXCL11 was presented to the cells together with FN, especially in the first 6 hr (Figure 5B, 5C). The presence of Fg together with CXCL11 did not enhance keratinocyte migration compared to cells migrating towards CXCL11 alone (Figure 5B, 5C). Curve interpolation analysis was also performed to investigate the speed of migration during the first 6 hr. The highest speed of migration was detected when cells were migrating towards CXCL11/FN, at 240 ± 15 cells/hr compared to 73 ± 21 cells/hr observed when cells were migrating towards CXCL11 alone (Figure 5D), thus highlighting a possible role of FN in coordinating keratinocytes migration by properly presenting CXCL11 to the cells. FN alone did not enhanced keratinocyte transmigration or migration speed (Figure 5B, 5C, 5D). To test whether and which integrins were involved in the enhancement of keratinocyte migration observed when CXCL11 was presented to the cells together with FN, we blocked α5β1 and αVβ3 by using specific antibodies (Figure 5E). α5β1 and αVβ3 were chosen because they are two major receptors of FN. In the first 6 hr, blocking α5β1 and αVβ3 integrins did not affect keratinocyte migration towards CXCL11 alone, however blocking α5β1 integrin when CXCL11 was presented together with FN decreased keratinocyte migration to the level observed for migration in response to CXCL11 alone. αVβ3 blocking did not affect keratinocyte migration towards CXCL11 presented on FN. Statistical analysis by using a one-way ANOVA with Dunnett’s comparison to Control was performed and demonstrated the significance indicated above. 

### Co-delivery of CXCL11 and fibronectin enhances wound repair in diabetic mice

The enhancement of keratinocyte migration observed in the presence of CXCL11 with FN encouraged us to explore whether their co-delivery could enhance skin wound healing *in vivo*. Diabetic *db/db* mice, a genetic mouse model of type-2 diabetic mellitus, were used a as well established and widely used experimental model of delayed wound healing. Full-thickness excisional skin wounds were treated with fibrin functionalized with FN and containing CXCL11. Four different groups (n=8) were tested: fibrin only, fibrin containing CXCL11, fibrin functionalized with FN and fibrin functionalized with FN and containing CXCL11. The wounds were harvested after 10 d, and histological analysis was performed to analyze wound closure and granulation tissue formation. As statistical analysis, one-way ANOVA with Dunnett’s comparison to the fibrin group was performed. 

**Figure 6:**
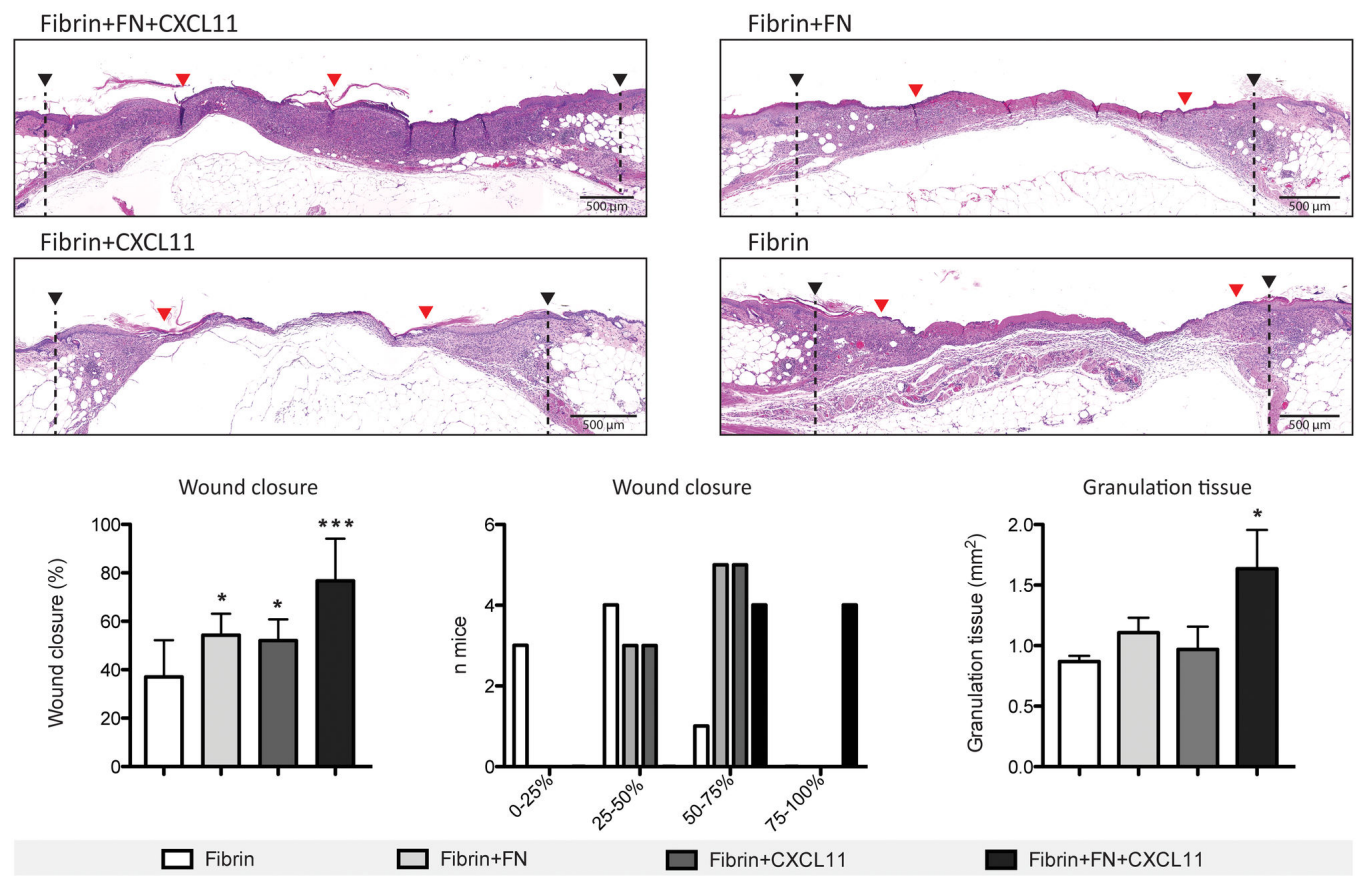
Wound healing in *db/db* mice. CXCL11 enhances wound healing when delivered in FN-functionalized fibrin matrices. Wound closure and granulation tissue formation was evaluated in full-thickness excisional skin wounds in diabetic *db/db* mice. Wound edges (black arrows) and tips of migrating epithelial tongues (red arrows) are indicated. (Representative images, n=8, mean ± SD, One-way ANOVA with Dunnett’s comparison to Fibrin group, * p<0.05, *** p<0.001).

**Figure 7:**
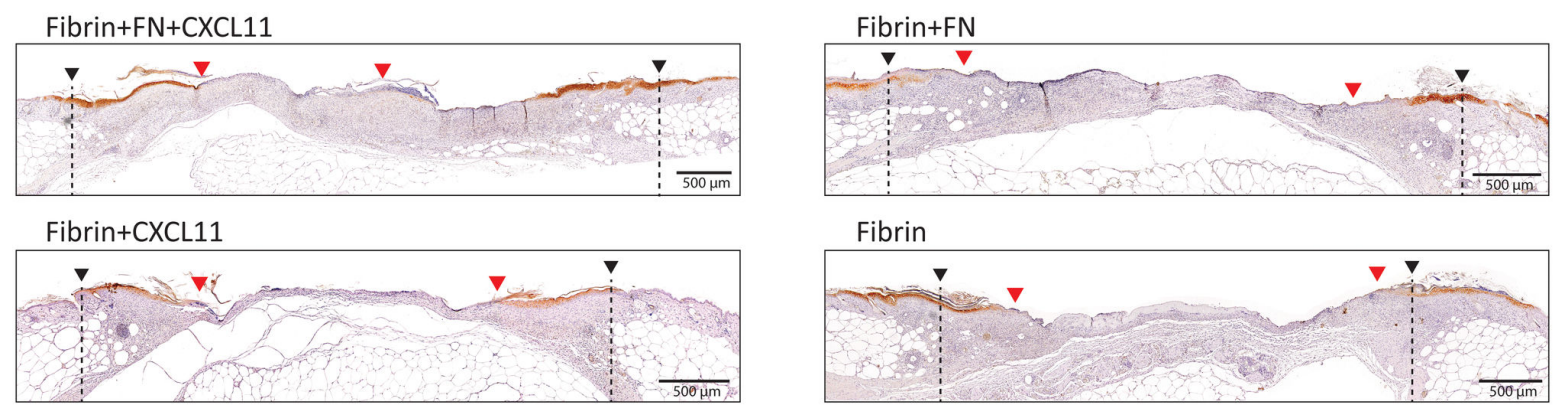
Cytokeratin 16 expression. Immunohistochemical analysis for cytokeratin 16 highlights hyper-proliferating keratinocytes in migrating epithelial tongues. Wound edges (black arrows) and tips of migrating epithelial tongues (red arrows) are indicated. Higher resolution images of epithelial tongue tips are shown in Figure S3. (Representative images, n=8, mean ± SD, One-way ANOVA with Dunnett’s comparison to Fibrin group, * p<0.05, *** p<0.001).

As shown in Figure 6, despite a moderate acceleration in wound closure, no statistically significant differences in granulation tissue formation were observed when wounds were treated with either fibrin functionalized with FN or fibrin containing CXCL11 compared to fibrin only-treated wounds. In contrast, delivery of CXCL11 in FN-functionalized fibrin led to a further increase in reepithelialization and to a two-fold higher amount of granulation tissue development (Figure 6). Indeed, high or complete (75% - 100%) wound closure by the 10th day was observed only in wounds treated with FN-functionalized fibrin containing CXCL11, while all the other treatments led to partial (25% - 75%) or almost absent (0% - 25%) wound closure. As expected, fibrin only treatment led to the least granulation tissue formation and slowest re-epithelialization. Immunohistochemical analysis for cytokeratin 16, a marker expressed by hyper-proliferating keratinocytes after wounding, further highlighted the differences in re-epithelialization due to the different treatments (Figure 7) and showed the tips of migrating epithelial tongues (Figure S3). Since both CXCR3 ligands and FN are important mediators in recruiting immune cells at sites of inflammation and since macrophages are important mediators of wound healing, we evaluated the presence of monocytes/macrophages in the granulation tissue by immunohistochemical analysis for CD68. Although we observed a higher amount of granulation tissue formation in wounds treated with FN-functionalized fibrin containing CXCL11 (Figure 6), no increased monocyte/macrophage infiltration was observed compared to wounds treated with either FN-functionalized or CXCL11-containing fibrin gels (Figure S3). 

## Discussion

Our group has previously investigated growth factor binding to FN[9,10] and Fg[16]. We have reported that binding of a growth factor to FN can modulate its signaling for the cases of vascular endothelial growth factor-A (VEGF-A), platelet-derived growth factor-BB (PDGF-BB) and bone morphogenetic protein-2 (BMP-2)[9]. Here, we turned our attention to chemokine-ECM interactions, and we show that CXCL10 and CXCL11, but not CXCL9, bind to FN and Fg, and further that FN but not Fg binding modulates CXCL11's biological activity toward keratinocyte migration. The interactions between CXCL11 and FN is particularly interesting in the context of wound healing, due to the role of CXCL11 in coordinating re-epithelialization[5,7] and to the importance of FN in controlling both cell migration through integrin signaling and growth factor partitioning, diffusion and signaling[9,20,21]. Here we showed that the effects of CXCL11 on *in vitro* keratinocyte migration and re-epithelialization *in vivo* are enhanced in the presence of FN, highlighting the importance of investigating the interactions between soluble morphogens (both growth factors and cytokines) and ECM molecules to both elucidate biological function in the physiological context and develop new biomatrices for regenerative medicine applications. 

Indeed, an emerging interest in cytokine delivery to mediate wound healing has grown in the last few years due to a better understanding of the complex integration of signals derived from the immune system and the regenerative microenvironment[2,22]. Although interactions between heparan sulfate glycosaminoglycans and several cytokines have been investigated[23,24], cytokine binding to protein components of the provisional matrix deposited during wound healing has been poorly investigated. We here sought to identify potential new interactions between CXCR3 ligands (i.e. CXCL9, CXCL10 and CXCL11) and two of the most important proteins of the provisional matrix deposited during tissue repair, FN and Fg. These two molecules were selected because of their clear role in acting as a reservoir for heparin-binding growth factors and in coordinating wound healing. Thus, we found that CXCL10 and CXCL11 bound to both FN and Fg (Figure 1), in addition to other immune regulators, especially IFN-γ (Figures S1 and S2). 

To better investigate the nature of the observed interactions, we then examined whether CXCL10 and CXCL11 bind the heparin binding domains of FN and Fg. Although FNIII 12-14 is a promiscuous growth factor binding domain, we observed no binding between it and CXCL10 or CXCL11, thus suggesting the involvement of another FN domain in the interactions. To this point, we found that CXCL10 and CXCL11 bound to the N-terminal heparin binding domain of FN (FNI 1-5) as well as to the heparin binding domain of Fg (Fg β15-66) with affinities (K_d_) in the nanomolar range (Figure 3), comparable with the affinities previously reported for growth factor binding to FNIII 12-14 and Fg β15-66[10,16]. We then showed that the presence of heparin decreased binding to both FNI 1-5 and Fg β15-66 (Figure 4), an opposite effect compared to the one observed for growth factor binding to FN[25,26]. These observations highlight the complexity and the differences between cytokine and growth factor binding to FN and Fg and suggest a possible role for specific molecular structures in mediating binding to ECM. 

As was observed to be the case for growth factor binding to Fg β15-66[16], the presence of a heparin binding domain within the cytokines was necessary, but not sufficient, to provide binding to both FNI 1-5 and Fg β15-66. Indeed, among the three tested CXCR3 ligands, only CXCL10 and CXCL11 bound FN and Fg, while they all bind heparin with different affinities[27-30]. CXCL9 has an extended highly positively charged C-terminus, compared to CXCL10 and CXCL11, which might be the responsible for the different binding behavior. Indeed, CXCL9 presents 27 and 30 extra amino acid residues at the C-terminus compared to CXCL10 and CXCL11, respectively, which have a short and highly similar C-terminal residues after Cys74, a conserved cysteine residue involved in a disulfide bond. Interestingly, the presence of cleavage sites for gelatinase B and neutrophil collagenase within this extended region[31] could suggest a possible change of CXCL9 binding properties following C-terminal cleavage. However, further studies are needed, and the nature of cytokine-ECM interactions will be likely understood in the future only by crystallographic analysis of the different complexes.

Since binding of FN to VEGF-A, PDGF-BB and BMP-2 was observed in previous studies from our laboratory to modulate the function of these growth factors [9], we turned our attention to the biological implications of FN and Fg binding to CXCL11. In particular, binding of CXCL11 to FN might be important during wound healing and re-epithelialization, where both molecules coordinate keratinocyte migration during tissue repair. FN can be crosslinked to fibrin within the provisional matrix by the coagulation transglutaminase factor XIIIa, and it is considered an important molecule in driving keratinocyte migration through several integrins, including α5β1, αvβ1 and αvβ6[32,33]. CXCL11 is produced by keratinocytes in response to damage and regulates re-epithelialization by enhancing their motility[6,7]. 

Here, we found that FN but not Fg binding modulates CXCL11's effects on keratinocyte migration in a transmigration *in vitro* assay, CXCL11-induced migration being strongly enhanced in the presence of FN. Blocking α5β1 integrin when CXCL11 was presented together with FN, but not αvβ3, decreased keratinocyte migration to the level observed for CXCL11 alone, suggesting involvement of this integrin in enhancing keratinocyte migration. The existence of an allosteric network among FNI 1-5, FNIII 12-14 and FNIII 9-10, the major α5β1 binding site of FN, together with recent findings showing that ligation of FNIII 10 increases accessibility of FNI 1-5 [34], suggest that the physical proximity of CXCR3 and α5β1 might be important for mediating induced keratinocyte migration. Notably, the absence of an allosteric network between Fg β15-66 and α5β1 binding sites in Fg [35] might explain the inability of Fg binding to CXCL11 in modulating CXLC11 activity. 

To evaluate the biological effects of FN binding *in vivo*, we used a full-thickness excisional wound model in *db/db* diabetic mice, a model commonly used during preclinical evaluation of therapeutics for chronic wound healing[9]. A significant enhancement of granulation tissue formation together with faster wound re-epithelialization and closure were observed in wounds treated with FN-functionalized fibrin matrices containing CXCL11, thus confirming an important role of FN and CXCL11 interactions in coordinating re-epithelialization and wound healing. Although our results suggest that binding to FN within the provisional matrix enhances re-epithelialization by enhancing keratinocyte migration, further experiments aiming at the study of the initial phases of wound healing are needed to better explore the effects of these interactions on granulation tissue formation. Indeed, despite increased granulation tissue formation in wounds treated with FN-functionalized fibrin gels containing CXCL11, the presence of monocyte/macrophages in the granulation tissue was not influenced by CXCL11 binding to FN, suggesting that binding does not likely influence granulation tissue composition. However, since CXCR3 ligands are important mediators in recruiting immune cells at inflammation sites[36] we can hypothesize that binding to FN might influence the initial stages of wound healing, potentially favoring both a broad immune cell infiltration and keratinocyte migration. 

Although CXCR3 ligands have been previously reported as important mediators of re-epithelialization[5,7], their use as therapeutic molecules in wound healing is, to our knowledge, unexplored. Our results indicate that they are potentially interesting molecules as therapeutic candidates, at least when the biomolecular interactions in the tissue or biomatrix microenvironment are well considered. More generally, our results highlight that better knowledge of the interactions occurring between growth factors, cytokines and the ECM will yield design rules by which appropriate partitioning and presentation of these important therapeutic molecules can be recreated. 

## Materials and Methods

### Ethics statement

All animal procedures and manipulations were conducted according to the ethical principles and guidelines for housing and experiments on animals of the Swiss federal veterinary office. The animal protocol describing the procedures was reviewed and approved by the veterinary affair office of the canton of Vaud, Switzerland.

### Cytokines, FNI 1-5 and Fg β15-66

All cytokines were purchased highly pure, carrier free and lyophilized from PeproTech EC Ltd. All cytokines were reconstituted and stored according to the manufacturer’s instructions. Full length FN from human plasma was purchased from Sigma-Aldrich. Human Fg depleted from plasminogen, vWF and fibronectin was purchased from MILAN Analytica AG.

FNIII 12-14 and Fg β15-66 were produced and purified as previously described[10,16]. FNI 1-5 (residues P51 to H273) was expressed in mammalian cells, HEK-293E, using the vector pXLG, which uses an IgK leader sequence for secretion of the FN fragment. NotI and BamHI restriction sites were used to clone the construct in frame. At the C-terminus of the construct is a short linker sequence, LE, followed by a 6×His tag sequence. HEK-293E cells were transfected with 1.25 μg of plasmid per 10^5^ cells per 1 mL of final growth medium (Excell 293, 4 mM glutamine, 3.75 mM valproic acid). The culture medium was harvested after 7 d of shaker flask expression and cells were removed by filtration. The protein was then purified using an FPLC (Akta Explorer, GE Healthcare) with a HisTrap HP column (GE Healthcare). The lipopolysaccharide (LPS) levels were detected with a HEK-BlueTM LPS Detection Kit (InvivoGen) to ensure no LPS presence in the fragments. 

### Detection of cytokine binding to ECM molecules

ELISA plates (Nunc MaxiSorp; Thermo Fisher Scientific) were coated with 50 nM cytokines (overnight at 4°C) and blocked with 2% BSA in PBS-Tween 20 (PBS-T, 0.05%) for 1 h at room temperature. Then, wells were washed with PBS-T and further incubated with of FN (10 nM), Fg (10 nM), FNI 1-5 (100 nM) or Fg β15-66 (100 nM), 30 min in PBS-T with 0.1% BSA). After 3 washes with PBS-T, wells were incubated with anti-FN (clone 5G7, Abcam), anti-Fg (Ab7539, Abcam) or anti-6xHis antibody conjugated to horseradish peroxidase accordingly to the tested molecule for 0.5–1 h. After washing, the antibody was detected with tetramethylbenzidine substrate measurement of the absorbance at 450 nm. Binding of FN, Fg, FN I1-5 and Fg β15-66 to BSA and binding of anti-FN, anti-Fg and anti-6xHis antibodies to the tested cytokines were considered as background and thus properly subtracted from binding results. PDGF-BB was used as positive binding control and absorbances were calibrated accordingly. 

### Heparin competition assay

Indirect ELISA was performed as described above, probing with either FNI 1-5 or Fg β15-66 in presence of different concentrations (2 nM - 20μM) of heparin (Sigma-Aldrich).

### *K*_d_ analysis

ELISA plates (Nunc MaxiSorp; Thermo Fisher Scientific) were coated with 20 nM CXCL10, 20 nM CXCL11 or 50 nM IFN-γ (overnight at 4°C) and blocked with 2% BSA in PBS-Tween 20 (PBS-T, 0.05%) for 1 h at room temperature. Then, wells were washed with PBS-T and further incubated with increasing concentrations (25 nM - 3.2 μM) of either FNI 1-5 or Fg β15-66 for 30 min in PBS-T with 0.1% BSA). After 3 washes with PBS-T, wells were incubated with anti-6xHis antibody conjugated to horseradish peroxidase for 0.5–1 h. After washing, the antibody was detected with tetramethylbenzidine substrate measurement of the absorbance at 450 nm. Binding to BSA-coated wells was used as control and the absorbance subtracted from the aborbance obtained from cytokines-coated wells. K_d_ were then calculated by linearization of the curves as described elsewhere[19].

### Modified-Boyden chamber transmigration assay and time lapse image analysis

Chemotaxis was studied by real time transwell migration assay using 8 μm-pore inserts (BD Falcon Fluoroblok) in a 24-well plate format. HeCaT cells, adherent in a cell culture flask, were stained with 4 μl/mL of Vybrant® DIO cell tracer (Life Technologies Corp.) in serum free medium. After washing extensively with complete medium, cells were detached with accutase (Biological Industries), resuspended in serum free DMEM or with blocking antibodies for either α5β1 (Abcam clone J8S5, 1μg/ml or αvβ3 (Abcam clone 23C6, 1μg/ml) and then placed in the top chamber of the inserts (1 x 106 cells in 150 μl). Equimolar concentrations of CXCL11 (0.2 μg/ml), FN (5 μg/ml) and Fg (7 μg/ml) were added in the bottom chamber accordingly to the experiment in 700 μl of serum free DMEM per insert. The plates were sealed, connected to 5% CO2-Air mix, placed at 37°C in the Cell IQ MLF® live imaging microscope (CM Technologies Oy, Tampere, Finland) and fluorescence images of the lower side of the insert membranes were acquired for 12 h at 15 min intervals using a 10x objective. Transmigration was quantified through automatic cell identification and cell counting using the CellIQ Analyser® software (CM Technologies, Tampere, Finland). Segmented interpolation analysis was performed to investigate the speed of migration during the first 6 hr.

### Full-thickness excisional skin wound model

C57BLKS/J-m/Lepr db (*db/db*) male mice were 10 to 12 wk old at the start of the experiments. Their backs were shaved and four full-thickness punch biopsy wounds (6 mm in diameter) were created in each mouse. Directly after, fibrin matrices [80 μl total, fibrinogen (10 mg/ml), 0.5 μM FN, 50 ng murine CXCL11] were polymerized on the wounds. To avoid animal- and position-dependency each animal was treated with the four tested fibrin matrices in randomized positions. To avoid drying of the matrices, the wounds were covered with non-adhering dressing (Adaptic, Johnson & Johnson) and then with adhesive film dressing (Hydrofilm, Hartmann). After 10 d the animals were sacrificed and the wounds were harvested for histological analysis. An area of 10 mm in diameter, which includes the complete epithelial margins, was excised. Wounds were cut in the middle and embedded. Histological analysis was performed on serial sections (4-μm paraffin sections) starting from the central part of the wound. Wound closure and granulation tissue formation were measured by histomorphometric analysis of tissue sections (hematoxylin and eosin stain) by using ImageJ software. Wound edges were defined by the position of the panniculus carnosus edges and by the morphology of unwounded epidermis. Wound closure was defined by looking at tips of migrating epithelial tongue and by measuring the percentage of the wound that was covered with new epidermis. Granulation tissue area was defined as the area of highly cellularized tissue observed within the wound bed. 

### Immunohistemical analysis

Paraffin-embedded tissue sections were dewaxed, rehydrated and endogenous peroxidase quenched with 3% H_2_O_2_ solution in PBS. A antigen retrieval step at 95°C for 20 min in 10 mM Tris-Citrate buffer was performed before blocking with 2% BSA solution in PBS for 1 hr. Sections were then incubated overnight at 4°C with primary antibodies against either Cytokeratin 16 (Abcam ab53117, 1:200 in 2% BSA solution in PBS) or CD68 (Abcam ab125212, 1:100 in 2% BSA solution in PBS). HRP-conjugated secondary anti-rabbit antibody (Dako) and SIGMA*FAST* ^TM^ DAB (Sigma-Aldrich) were used.

## Supporting Information

Figure S1 **Fibronectin binds cytokines from different families.** Binding to FN was determined by indirect ELISA and calibrated to PDGF-BB binding as a strongly binding reference (Abs 450nm = 0.59 AU). FN binding to BSA was considered as background and subtracted. Binding of IL-2, IL-4, IFN-γ, CCL20 was observed (*). (n=6, mean ± SD).
(TIF)

Figure S2 **Fibrinogen binds cytokines from different families.** Binding to Fg was determined by indirect ELISA and calibrated to PDGF-BB binding as a strongly binding reference (Abs 450nm = 0.79 AU). Fg binding to BSA was considered as background and subtracted. Binding of IL-1α, IFN-β and IFN-γ was observed (*). (n=6, mean ± SD).
(TIF)

Figure S3 **Cytokeratin 16 and CD68 immunohistochemical analysis.** Cytokeratin 16 and CD68 positive cells were detected by immunohistochemical analysis with DAB staining and Meyer’s hematoxylin counterstaining. High magnification representative images of the tips of migrating epithelial cells (cytokeratin 16) and granulation tissue (CD68) are shown. (Scale bars, 100 μm).
(TIF)
