# Supplementary figures and images for: Fibronectin Binding Modulates CXCL11 Activity and Facilitates Wound Healing
Source: PLoS One. 2013 Oct 25;8(10):e79610. doi: 10.1371/journal.pone.0079610 (PMC3808276; doi:10.1371/journal.pone.0079610)

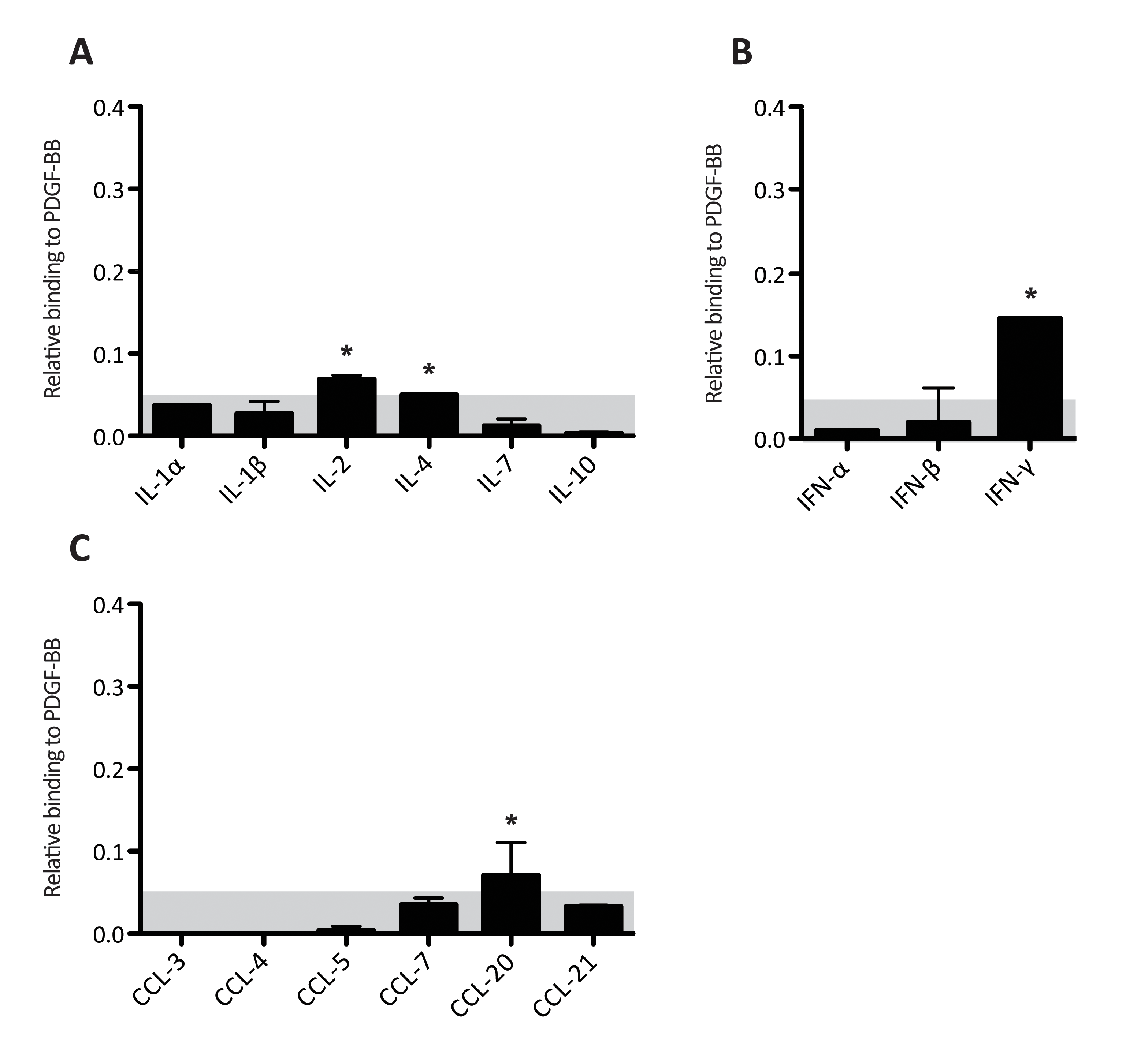

Supplement: Figure S1 — Fibronectin binds cytokines from different families. Binding to FN was determined by indirect ELISA and calibrated to PDGF-BB binding as a strongly binding reference (Abs 450nm = 0.59 AU). FN binding to BSA was considered as background and subtracted. Binding of IL-2, IL-4, IFN-γ, CCL20 was observed (*). (n=6, mean ± SD). (TIF) [file pone.0079610.s001.tif]

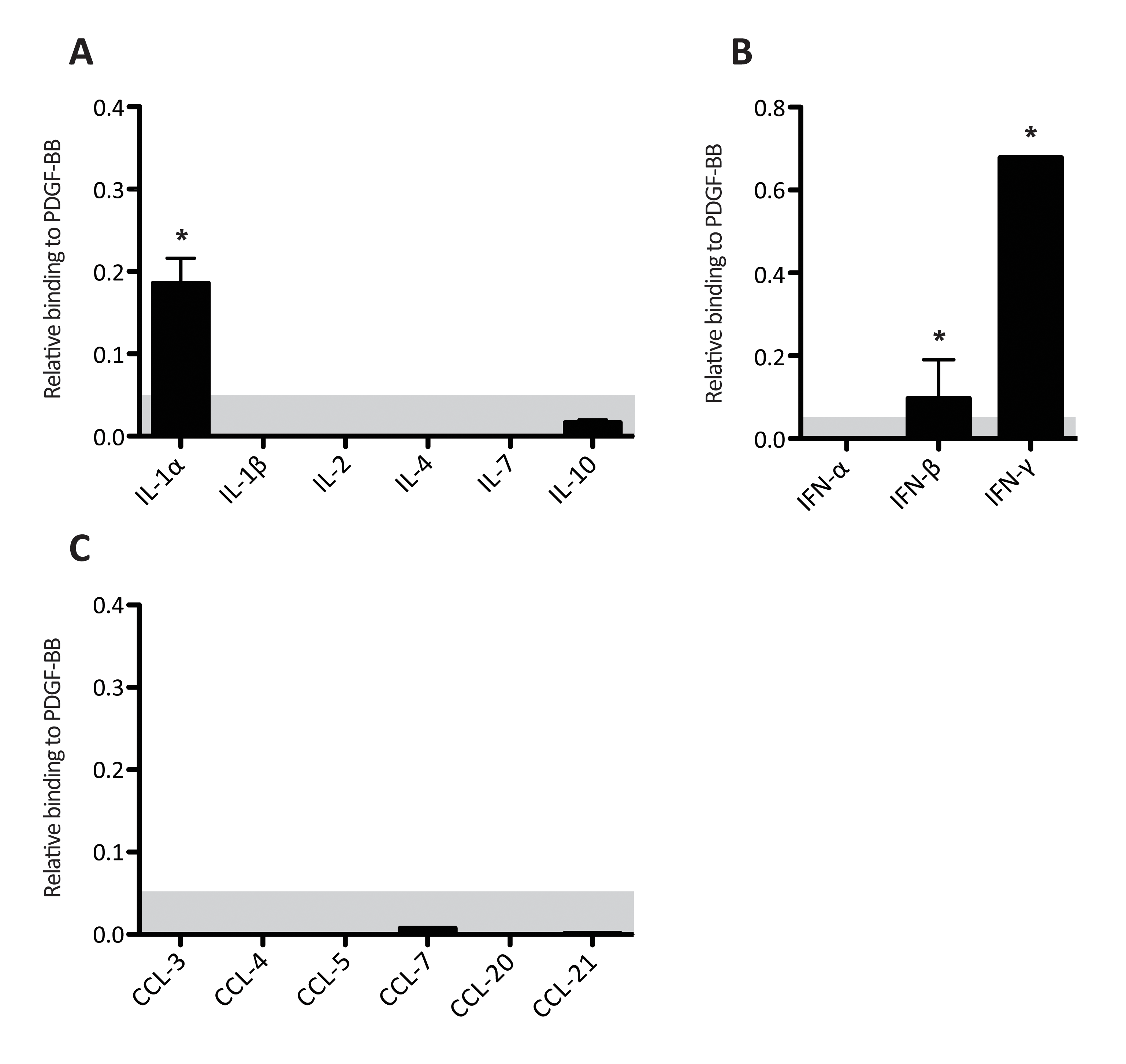

Supplement: Figure S2 — Fibrinogen binds cytokines from different families. Binding to Fg was determined by indirect ELISA and calibrated to PDGF-BB binding as a strongly binding reference (Abs 450nm = 0.79 AU). Fg binding to BSA was considered as background and subtracted. Binding of IL-1α, IFN-β and IFN-γ was observed (*). (n=6, mean ± SD). (TIF) [file pone.0079610.s002.tif]

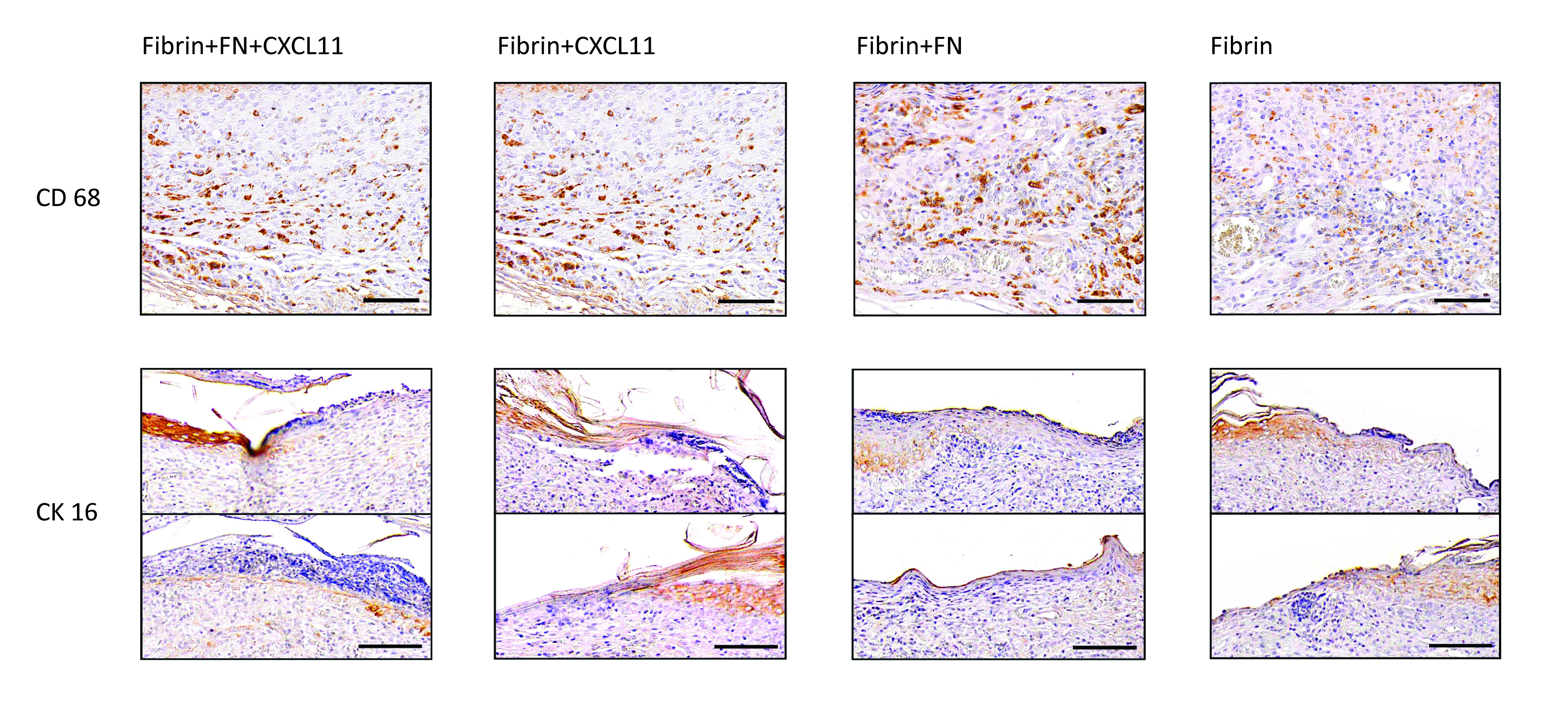

Supplement: Figure S3 — Cytokeratin 16 and CD68 immunohistochemical analysis. Cytokeratin 16 and CD68 positive cells were detected by immunohistochemical analysis with DAB staining and Meyer’s hematoxylin counterstaining. High magnification representative images of the tips of migrating epithelial cells (cytokeratin 16) and granulation tissue (CD68) are shown. (Scale bars, 100 μm). (TIF) [file pone.0079610.s003.tif]
